# Efficacy and safety of accelerated deep TMS for depressed patient with active suicidality

**DOI:** 10.1192/j.eurpsy.2025.1319

**Published:** 2025-08-26

**Authors:** K. Feffer, E. Bloemhof-Bris, Y. Levi-Belz, A. Tamari-Guterman, G. Wexler, Y. Ben-Ari, M. Rachman, S. Noam

**Affiliations:** 1 Lev Hasharon Mental Health Center, Tsur Moshe; 2 Faculty of Medical and Health Sciences, Tel Aviv University, Tel Aviv; 3 The Lior Tsfaty Center for Suicide and Mental Pain Studies, Ruppin Academic Center, Emek Hefer, Israel

## Abstract

**Introduction:**

Suicidality is one of the leading causes of death in young adults. Most of the suicidal patients suffer from background depression. Developments in the last few years in brain simulation technology as dTMS enables a more intensive care in accelerated protocols that have already been approved in the treatment of the depression field. However, most major studies in the field excluded the participation of patient with active suicidality.

**Objectives:**

This study focuses on efficacy of dTMS in patients with active suicidality.

**Methods:**

This double-blind randomized study offers an accelerated protocol dTMS treatment in the span of 10 treatment days, while the patient is maintained in full hospitalization and secure conditions. The study examined the efficacy of the dTMS accelerated protocol (which includes 3 treatments a day for the course of 10 treatment days) on suicidality indicators. This treatment was given in addition (add-on) to standard ward treatments in three arms (1:1:1) sham, active H1 coil and active H7 coil.

**Results:**

38 patients were enrolled in the study. 30 patients successfully completed 10 treatment days. 8 patients did not complete treatment due to withdraw of informed consent before starting treatment (N = 4), panic attack (N = 2), discharge from the ward (N = 1) and suicide attempt (N = 1). Patients received active treatment (both H1 and H7) showed superior response (defined as >50% improvement in depression scale) compared to placebo arm (PA) (p = 0.03). Additionally, clinically close to significance improvement in suicidality intensity scales was found after 5 and 10 days of treatment in active treatment compared to placebo (p = 0.09). However, no significant difference was found regarding suicidality type scores. No major differences in depression and suicidality were found comparing H1 and H7 coils. The main side effects were headaches and dizziness, motoric tremor during the treatments, vomiting and general exhaustion. One patient (placebo group) completed suicide a month following the end of the study.

**Conclusions:**

Active suicidality is a major challenge in treating severe mental disorders, and death by suicide is still a leading cause of death among patients. However, most clinical studies in mental health still exclude patients with active suicidality, hence treatment options are limited. In this study, we found deep TMS accelerated protocol (both H1 and H7 coils) to be both safe and effective in decreasing suicidality intensity and depression symptoms among inpatients with severe mental disorders accompanied by suicidality. Further studies are needed to differentiate H1 and H7 coils effectiveness in different depression sub-types and common psychiatric comorbidities.

**Disclosure of Interest:**

None Declared

